# Joint effects of carotid plaques and renal impairment on the risk of cardiovascular disease and all-cause death in a community-based population: The Kailuan cohort study

**DOI:** 10.3389/fcvm.2022.943718

**Published:** 2022-11-17

**Authors:** Wen Li, Wenkun Bai, Congliang Miao, Shuohua Chen, Xinyu Zhang, Yanfeng Fan, Xiao Li, Shouling Wu, Xuemei Liu, Jiang Hong

**Affiliations:** ^1^Department of Ultrasound in Medicine, Shanghai Sixth People’s Hospital Affiliated to Shanghai Jiao Tong University School of Medicine, Shanghai, China; ^2^Department of Internal and Emergency Medicine, Shanghai General Hospital, Shanghai Jiao Tong University School of Medicine, Shanghai, China; ^3^Department of Cardiology, Kailuan General Hospital Affiliated to North China University of Science and Technology, Tangshan, China; ^4^Department of Ultrasound in Medicine, Beijing Hospital of Traditional Chinese Medicine, Capital Medical University, Beijing, China

**Keywords:** carotid plaque, estimated glomerular filtration rate, cardiovascular disease, all-cause death, community-based populations

## Abstract

**Objective:**

It is unknown whether renal impairment and atherosclerosis increase the risk of cardiovascular disease (CVD) and death. Atherosclerosis already raises the risk of CVD and all-cause death. This study investigated the joint effects of carotid plaques and renal impairment on CVD and all-cause death in community-based populations.

**Methods:**

The study cohort consisted of 20,416 participants from the Kailuan Study who completed a carotid plaque ultrasound in 2012. A glomerular filtration rate (GFR) of < 60 ml/min or trace semiquantitative proteinuria or higher were both considered signs of renal insufficiency. We divided them into four groups according to the presence of carotid plaque and renal impairment. These groups were categorized as no carotid plaque, estimated glomerular filtration rate (eGFR) ≥ 60 ml/min, and proteinuria < trace; no carotid plaque, eGFR < 60 ml/min, and proteinuria ≥ trace; carotid plaque, eGFR ≥ 60 ml/min and proteinuria < trace; and carotid plaque, eGFR < 60 ml/min, and proteinuria ≥ trace, respectively. We investigated the combined effect of renal impairment and carotid plaque on cardiovascular events and all-cause death in the Kailuan community-based population.

**Result:**

Participants with carotid plaque, eGFR < 60 ml/min and proteinuria had a 2.88-fold higher risk of all-cause death (95% CI, 2.18–3.80), which was significantly higher than those with lone factors (HR, 1.57; 95% CI, 1.04–2.36; and HR, 1.91; 95% CI, 1.56–2.32), compared to participants with no carotid plaque, eGFR ≥ 60 ml/min and proteinuria <trace group. Participants with carotid plaque, eGFR < 60 ml/min, and proteinuria had a 1.05-fold higher risk of CVD (95% CI, 0.82–1.35), which was not higher than those with alone factors (HR, 1.35; 95% CI, 1.02–1.80; and HR, 1.12; 95% CI, 0.96–1.30), compared to participants with no carotid plaque, eGFR ≥ 60 ml/min and proteinuria <trace group. Stratified analysis by age, participants with the carotid plaque, eGFR < 60 ml/min and proteinuria had a 2.98-fold higher risk of all-cause death (95% CI: 2.24–3.96), which was significantly higher than participants with lone factors (HR, 1.68; 95% CI, 1.10–2.59; and HR, 1.95; 95% CI, 1.59–2.40), compared to participants with no carotid plaque, eGFR ≥ 60 ml/min and proteinuria <trace group in the age of ≥ 50 years. Participants with carotid plaque, eGFR < 60 ml/min and proteinuria had a 1.66-fold higher risk of CVD (95% CI: 1.29–2.25), which was significantly higher than participants with lone factors (HR, 1.63; 95% CI, 1.20–2.22, and HR, 1.28; 95% CI, 1.11–1.49), compared to participants with no carotid plaque, eGFR ≥ 60 ml/min and proteinuria <trace group, in the age of ≥ 50 years.

**Conclusion:**

The joint of carotid plaques and renal impairment may further increase the risk of CVD and all-cause death compared with participants with alone factors in the age of ≥ 50 years, but not in the age of < 50 years, from a community-based study.

## Introduction

Traditional risk factors, such as hypertension, hyperglycemia, high blood lipids, renal impairment, and atherosclerosis, are part of multiple risk factor clusters that can increase the risk of cardiovascular disease (CVD) death and all-cause death in European and Asian populations (1–4). In China, a high incidence of atherosclerosis (5) and renal impairment (6) have a high lethality with CVD (2, 3) that significantly affects public health. Finding joint risk factors and performing a mortality risk assessment for adverse events could benefit high-risk individuals.

Impaired estimated glomerular filtration rate (eGFR) and proteinuria are markers of renal impairment that increase the prevalence of heart failure (7) and mortality. The risk of all-cause death in patients with chronic kidney disease (CKD) increased by 18–214% when eGFR was reduced from 60 ml/min/1.73^2^ to 15 ml/min/1.73^2^ (8). Proteinuria (ACR or urine dipstick method) is associated with a doubled risk of all-cause death (9). Furthermore, a meta-analysis found that those with low eGFR and proteinuria had a higher risk of all-cause death (8).

The formation of atherosclerotic plaque is the hallmark of atherosclerosis, and plaque ruptures are the cause of ischemic cerebrovascular disease (10), with significantly increased rates of lethality. Many studies have found a significantly increased risk in the general population (3) for those who have carotid plaques, as reported in studies on impaired renal function (11). Therefore, atherosclerotic plaque presence has become an important indicator for cardiovascular risk assessment in clinical studies.

Carotid plaque and renal impairment are risk factors for all-cause death and CVD (1, 2), and studies have shown that CKD tends to aggravate atherosclerosis and vascular calcification (12). However, it is unknown how carotid plaque and renal impairment together affect all-cause death and CVD. We assumed that patients with both renal impairment and carotid plaque have a greater risk of all-cause death and CVD than patients with only one of these risk factors. We used the Kailuan Study data to look at the combined effects of carotid plaque and renal impairment on CVD and all-cause death to confirm the inference. As such, this study can contribute to the identification of patients at high risk of CVD or all-cause death in community-based populations.

## Materials and Methods

### Study design and population

The Kailuan Study is a community-based cohort study exploring risk factors for cardiovascular and cerebrovascular diseases. It was initiated in 2006–2007, involving 101,510 adults aged 18–98 years in the Kailuan community in Tangshan City, a northern industrial city in China (13). All participants underwent questionnaire assessments, clinical examinations, and laboratory assessments once every 2 years by employee investigators. In 2012, data on carotid plaques were collected from 20,988 people who had undergone carotid artery ultrasound examinations (16,370 men and 4,618 women). Participants with a history of myocardial infarction (MI), stroke, or missing data were excluded. In accordance with the Helsinki Declaration, the protocol was approved by the Ethics Committee of the Kailuan General Hospital [(2006) Approval No. 5], and all participants gave written informed consent to participate in the study.

### Data collection

Data on demographic characteristics were collected *via* standardized questionnaires in 2012, including age, sex, smoking, drinking, lifestyle, use of medications (e.g., hypoglycemic agents, and antihypertensives), and history of MI and stroke. Smoking was defined as currently smoking “yes” or “no” based on the participants’ self-reports. Alcohol consumption of ≥ 100 ml/day or more per day for more than a year was defined as drinking.

In 2012, a health professional collected weight and height during a physical examination; BMI was calculated as weight (kilogram)/height (square meters). Systolic blood pressure (SBP) and diastolic blood pressure (DBP) were measured twice in the seated position using a mercury sphygmomanometer and an average of the two readings was used for the analyses. Hypertension was defined as blood pressure ≥ 140/90 mmHg or self-reported use of antihypertensive drugs. The fasting blood glucose (FBG), total cholesterol (TC), low-density lipoprotein cholesterol (LDL-C), and high-density lipoprotein cholesterol (HDL-C) were measured by an enzymatic method using an autoanalyzer (Hitachi 747; Hitachi, Tokyo, Japan) at the central laboratory of the Kailuan General Hospital. Diabetes was defined as fasting blood glucose ≥ 126 mg/dl or taking pills or insulin for diabetes. Dyslipidemia was defined as TC ≥ 220 mg/dl, LDL-C ≥ 140 mg/dl, HDL-C ≤ 40 mg/dl, or self-reported use of lipid-lowering drugs.

Participants in some analyses were divided into subgroups based on age, sex, SBP, DBP, FBG, LDL-C, HDL-C, TC, and BMI. The age subgroups were 40–49 years and ≥ 50 years. The SBP subgroups were < 130 mmHg (normal), 130–139 mmHg, 140–159 mmHg, 160–179 mmHg, and ≥ 180 mmHg, and the DBP subgroups were < 85 mmHg (normal), 85–89 mmHg, 90–99 mmHg, 100–109 mmHg, and ≥ 110 mmHg (14). The FBG subgroups were < 6.1 mmol/L (hypoglycemia), 6.1–6.9 mmol/L (normoglycemia), and ≥ 7.0 mmol/L [hyperglycemia (14)]. The LDL-C subgroups were < 4.1 mmol/L (normal), and ≥ 4.1 mmol/L (higher), and the HDL-C subgroups were < 1.0 mmol/L (low), and ≥ 1.0 mmol/L [normal (14)]. The TC subgroups were < 6.2 mmol/L (normal) and ≥ 6.2 mmol/L (higher) (14). The BMI subgroups were < 24.0 kg/m^2^ (normal weight), 24.0–27.9 kg/m^2^ (overweight), and ≥ 28 kg/m^2^ (obese) (15).

### Assessment of estimated glomerular filtration rate and semiquantitative proteinuria

Overnight fasting (8–12 h) venous blood samples were collected before 9:00 a.m. at the physical examination in 2012. With a lower detection limit of 22 μmol/L and an upper detection limit of 3000 μmol/L [linear correlation coefficient of (0.99)], serum creatinine was measured using the sarcosine oxidase assay method (creatinine kit; BioSino Biotechnology and Science Inc., Beijing, China). The intra- and inter-assay variable coefficients for serum creatinine were ≤ 5% and ≤ 6%, respectively, within the laboratory. eGFR was computed using serum creatinine, sex, and age, according to the CKD Epidemiology Collaboration equation:


(1) $$\text{eGFR}=\text{141}\times \text{min}{\left(\text{SCr}/\text{κ},\text{1}\right)}^{\text{α}}\times \text{max }{\left(\text{SCr}/\text{κ},\text{1}\right)}^{-\text{1.}209}\times 0.{\text{993}}^{\text{age}}\times \text{1.}0\text{18}\left[\text{if female}\right]\text{ (16, 17).}$$

SCr is serum creatinine, κ is 0.7 for women and 0.9 for men, α is -0.329 for women and -0.411 for men, min (SCr/κ,1) was the minimum value between SCr/κ and 1, and max (SCr/κ,1) was the maximum value between SCr/κ and 1.

Proteinuria was detected using an automated dipstick urinalysis (H12-MA test strips; Changchun Dirui Medical Technology Co., Ltd., Changchun, China; N-600; Changchun Dirui Medical Technology Co., Ltd.). The urinalysis was performed on a fresh urine sample by 3 physicians and read visually for 1 min right after the dipstick test. The results of semiquantitative proteinuria were recorded as negative (< 15 mg/dl), trace (15–29 mg/dl), 1+ (30–300 mg/dl), 2+ (300–1000 mg/dl), or 3+ (> 1000 mg/dl) and we defined proteinuria as trace or greater amounts of protein. Renal impairment was defined as a glomerular filtration rate of less than 60 ml/min or trace or more semiquantitative proteinuria.

### Assessment of carotid plaques

According to the American Society of Echocardiography, carotid artery scanning was fully performed (18). Participants were examined in a supine position with mild head extension and underwent a bilateral carotid duplex ultrasound (Philips iU-22 Ultrasound System, transducer 11 MHz, Philips Medical Systems, Bothell, Washington) to evaluate the presence of carotid plaques. Carotid artery plaque was defined as a focal structure that encroaches into the arterial lumen at least 0.5 mm or 50% of the surrounding IMT value or demonstrates a thickness > 1.5 mm as measured from the media adventitia interface to the intima-lumen interface (19).

### The criteria for grouping

We divided the participants into four groups according to the presence or absence of carotid plaque and renal impairment. These groups were no carotid plaque, eGFR ≥ 60 ml/min and proteinuria < trace; no carotid plaque, eGFR < 60 ml/min and proteinuria ≥ trace; carotid plaque, eGFR ≥ 60 ml/min and proteinuria < trace; and carotid plaque, eGFR < 60 ml/min and proteinuria ≥ trace, respectively.

### Follow-up and outcomes

The incident CVD events (including MI and cerebral infarction) and all-cause death are the main outcomes of our study. The evaluation of incident CVD events and all-cause death has been detailed previously (20–22). Summarily, participants were followed using face-to-face interviews during routine medical examinations every 2 years until 31 December 2017. All-cause death was defined as death from any cause, which was confirmed by either a death certificate from the local citizen registry or the record maintained by the hospital providing treatment. Myocardial infarction was defined according to the World Health Organization Multinational Monitoring of Trends and Determinants in Cardiovascular Disease Project’s criteria (23). Cerebral infarction was diagnosed according to the World Health Organization (24) criteria based on combined neurological signs and symptoms and imaging examinations, including computed tomography scans or MRI reports as detailed previously (22). The Data Safety Monitoring Board and the Clinical Outcomes Arbitration Committee both approved all of the results.

### Statistical analyses

Baseline characteristics were presented in descriptive statistics, with mean ± SD given for the normally distributed (according to the Kolmogorov–Smirnov test) continuous variables. In order to compare the continuous variables between groups, an analysis of variance (ANOVA) was used. The categorical variables were determined using the Chi-square test. By calculating hazard ratios (HRs) and 95% confidence intervals, Cox proportional hazards regression was used to estimate the risk of events (CIs). The model was adjusted for age, sex, smoking, alcohol, BMI, FBG, TC, LDL-C, SBP, DBP, antihypertensive drug use, and lipid-lowering drug use. The Kaplan–Meier method and the log-rank test were used to compare the rates of outcome events across groups. All statistical tests were two-tailed; a *P*-value of 0.05 was considered statistically significant. The analyses were performed using SAS 9.3 (SAS Institute, Cary, NC, USA).

### Clinical trial registration

Chinese Clinical Trials Registry, ChiCTR-TNC-11001489 (retrospective registration).

## Results

### Baseline characteristics of the study population

**FIGURE 1:**
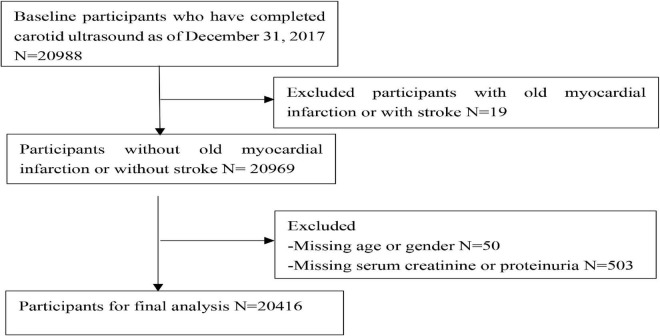
Flow chart of the study participants.

A total of 20,416 participants from the entire cohort (mean age, 53.65 ± 11.63 years, 78.3% of males) were analyzed. The flow chart is shown in Figure 1. There were significant differences between the groups for age, sex, smoking, drinking, FBG, SBP, DBP, TC, LDL-C, HDL-C, taking the antihypertensive drug, taking the hypoglycemic drug, and follow-up, the characteristics of which are presented in Table 1. The baseline characteristics stratified according to age, SBP, DBP, FBG, LDL-C, HDL-C, TC, and BMI are shown in Supplementary Table 1.

**TABLE 1:** Baseline characteristics of the study population.

Characteristics	No carotid plaque, eGFR ≥ 60 ml/min and proteinuria < trace *n* = 12602	No carotid plaque, eGF < 60 ml/min, and proteinuria ≥ trace *n* = 922	Carotid plaque, eGFR ≥ 60 ml/min, and proteinuria < trace *n* = 6023	Carotid plaque, eGFR < 60 ml/min, and proteinuria ≥ trace *n* = 869	All *n* = 20416	*P-value* for trend
Age (year)	50.46 ± 9.15	54.20 ± 11.85	61.89 ± 10.50	66.14 ± 10.37	56.18 ± 8.32	< 0.001
Male (%)	9590 (76.1)	638 (69.2)	5037 (83.6)	712 (81.9)	15977 (78.3)	< 0.001
Smoking (%)	5091 (40.4)	308 (33.4)	2632 (43.7)	353 (40.6)	8390 (41.1)	< 0.001
Drinking (%)	4196 (33.3)	274 (29.8)	2144 (35.6)	277 (31.9)	6900 (33.8)	< 0.001
FBG (mmol/L)	5.81 ± 1.66	5.97 ± 2.07	6.23 ± 2.19	6.16 ± 1.96	5.81 ± 1.66	< 0.001
SBP (mmHg)	134.63 ± 18.13	140.00 ± 21.87	143.43 ± 20.94	146.89 ± 22.32	142.32 ± 20.62	< 0.001
DBP (mmHg)	83.68 ± 10.79	87.11 ± 11.27	85.09 ± 11.25	85.14 ± 13.35	83.68 ± 10.79	< 0.001
BMI (kg/m^2^)	25.16 ± 3.37	25.75 ± 3.54	25.14 ± 3.23	25.21 ± 3.39	25.12 ± 3.44	0.488
TC (mmol/L)	5.15 ± 1.06	5.05 ± 1.05	5.28 ± 1.79	5.30 ± 1.21	5.21 ± 1.40	< 0.001
LDL-C (mmol/L)	2.92 ± 1.05	2.82 ± 0.81	3.00 ± 0.92	2.98 ± 0.94	2.99 ± 0.97	< 0.001
HDL-C (mmol/L)	1.44 ± 0.48	1.38 ± 0.36	1.51 ± 0.91	1.45 ± 0.40	1.47 ± 0.69	< 0.001
Taking antiplatelet drug (%)	50 (0.4)	11 (1.2)	101 (1.6)	42 (4.8)	204 (1.0)	< 0.001
Taking hypoglycemic drug (%)	252 (2.0)	45 (4.9)	373 (6.2)	80 (9.2)	750 (3.6)	< 0.001
Taking antihypertensive drug (%)	1071 (8.5)	147 (15.9)	1223 (20.3)	271 (31.2)	2712 (13.3)	< 0.001
Taking lipid-lowering drug (%)	264 (2.1)	43 (4.7)	361 (6.0)	104 (12.0)	772 (3.8)	< 0.001

BMI, body mass index; SBP, Systolic blood pressure; DBP, diastolic blood pressure; FBG, fasting blood glucose; HDL-C, high-density lipoprotein cholesterol; LDL-C, low-density lipoprotein cholesterol; TC, total cholesterol.

### All-cause death and cardiovascular disease events

**FIGURE 2:**
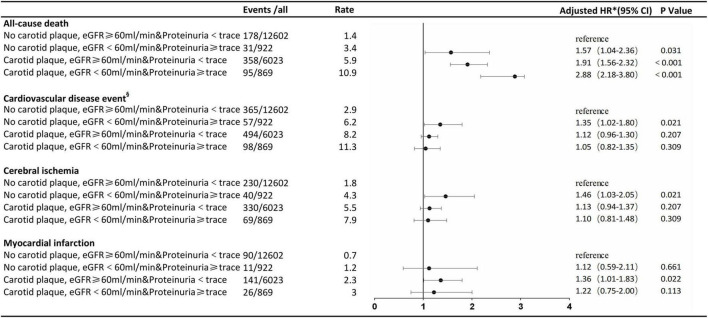
Association of carotid plaque and renal impairment with all-cause death and cardiovascular disease events. ^§^Cerebral ischemia or myocardial infarction. *The model was adjusted for age, sex, smoking, alcohol, body mass index, fasting blood glucose, total cholesterol, low-density lipoprotein-cholesterol, systolic blood pressure, diastolic blood pressure, antihypertensive drug use, and lipid-lowering drug use. eGFR, estimated glomerular filtration rate; HR, hazard ratio.

After a follow-up of 3.94 ± 2.02 years, we identified 662 all-cause deaths and 1,014 CVD. Adjusted for age, sex, smoking, alcohol, BMI, FBG, TC, LDL-C, SBP, DBP, antihypertensive drug use, and lipid-lowering drug use, participants with a carotid plaque, eGFR < 60 ml/min, and proteinuria had a 2.88-fold higher full-adjusted risk of all-cause death (95% CI, 2.18–3.80), which was significantly higher than those with lone factors (HR, 1.57; 95% CI, 1.04–2.36; and HR, 1.91; 95% CI, 1.56–2.32, respectively), compared to participants with no carotid plaque, eGFR ≥ 60 ml/min, and proteinuria <trace group. Participants with a carotid plaque, eGFR < 60 ml/min, and proteinuria had a 1.05-fold higher full-adjusted risk of CVD (95% CI, 0.82–1.35), which was not higher than those with lone factors (HR, 1.35; 95% CI, 1.02–1.80; and HR, 1.12; 95% CI, 0.96–1.30, respectively), compared to participants with no carotid plaque, eGFR ≥ 60 ml/min, and <trace proteinuria group, as shown in Figure 2.

### Age-specific subgroup analysis

**FIGURE 3:**
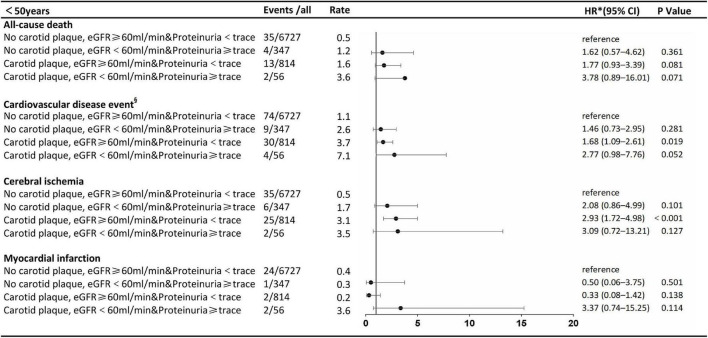
Association of carotid plaque and renal impairment with all-cause death and cardiovascular disease events in subgroups stratified according to age. ^§^Cerebral ischemia or myocardial infarction. *The model was adjusted for age, sex, smoking, alcohol, body mass index, fasting blood glucose, total cholesterol low-density lipoprotein-cholesterol, systolic blood pressure, diastolic blood pressure, antihypertensive drug use, and lipid-lowering drug use. eGFR, estimated glomerular filtration rate: HR, hazard ratio.

**FIGURE 4:**
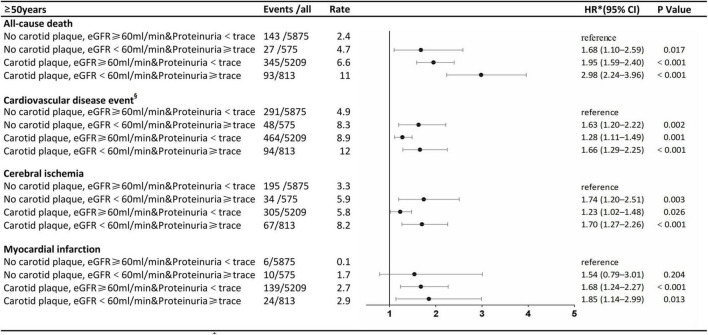
Association of carotid plaque and renal impairment with all-cause death and cardiovascular disease events in subgroups stratified according to age.

The stratified analysis by age for HRs of all-cause death and CVDs by renal impairment and carotid plaque is shown in Figures 3, 4. Participants with carotid plaque, eGFR < 60 ml/min, and proteinuria had a 2.98-fold higher full-adjusted risk of all-cause death (95% CI: 2.24–3.96), which was significantly higher than participants with lone factors (HR, 1.68; 95% CI, 1.10–2.59; and HR, 1.95; 95% CI, 1.59–2.40, respectively), compared to participants with no carotid plaque, eGFR ≥ 60 ml/min, and proteinuria <trace group in the age of ≥ 50 years. Participants with carotid plaque, eGFR < 60 ml/min, and proteinuria had a 1.66-fold higher full-adjusted risk of CVD (95% CI: 1.29–2.25), which was significantly higher than participants with lone factors (HR, 1.63; 95% CI, 1.20–2.22, and HR, 1.28; 95% CI, 1.11–1.49; respectively), compared to participants with no carotid plaque, eGFR ≥ 60 ml/min, and proteinuria <trace group, in the age of ≥ 50 years. However, participants under the age of 50 years did not show this trend.

### Kaplan–Meier analysis

**FIGURE 5:**
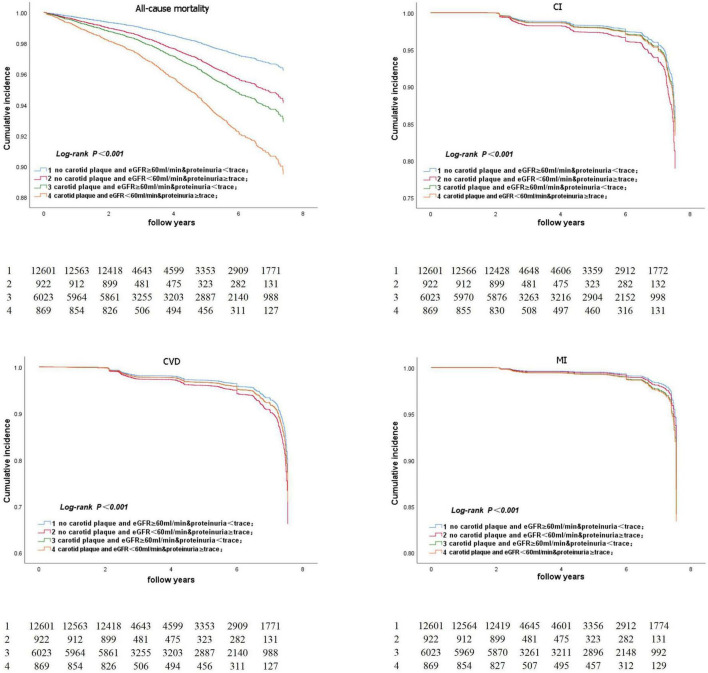
Kaplan-Meier survival curve for all-cause death and CVD event.

**FIGURE 6:**
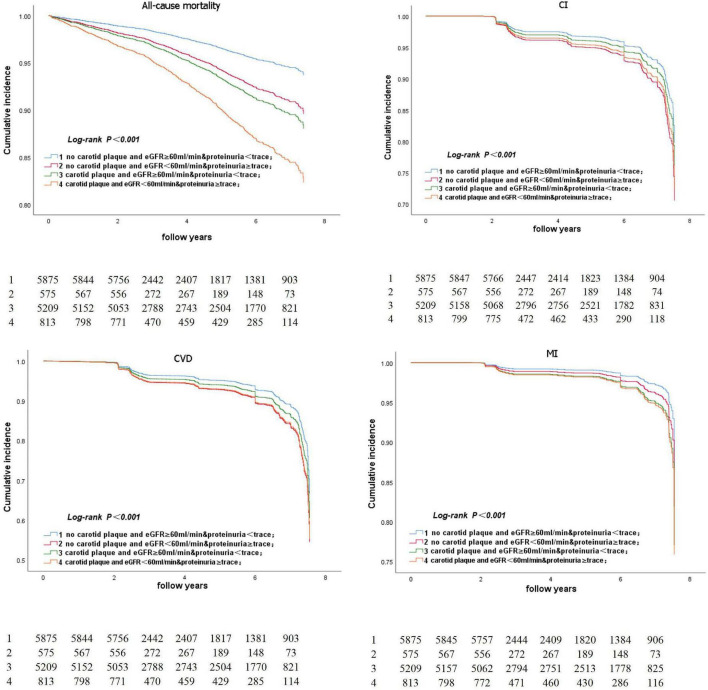
Kaplan-Meier survival curve for all-cause death and CVD event in participants aged ≥50 years.

Figure 5 represents the Kaplan–Meier survival curve for all-cause death and CVD events. The Kaplan–Meier survival curve for all-cause death and CVD events in participants aged ≥ 50 years is shown in Figure 6. The log-rank test revealed a significant difference (*p* < 0.001) between participants and two age subgroups.

### Gender-specific subgroup analysis

The stratified analysis by gender for HRs of all-cause death and CVDs by renal impairment and carotid plaque is in Supplementary Table 2. Male participants with carotid plaque, eGFR < 60 ml/min, and proteinuria had a 1.71-fold higher full-adjusted risk of all-cause death (95% CI: 1.15–2.54), which was significantly higher than participants with lone factors (HR, 1.45; 95% CI, 1.08–1.96; and HR, 1.16; 95% CI, 0.93–1.45, respectively), compared to participants in the no carotid plaque and eGFR ≥ 60 ml/min proteinuria <trace group. Male participants with a carotid plaque, eGFR < 60 ml/min, and proteinuria had a 1.35-fold higher full-adjusted risk of CVD (95% CI: 1.04–1.75), which was significantly higher than participants with lone factors (HR, 1.35; 95% CI, 0.97–1.87, and HR, 1.15; 95% CI, 0.97–1.35; respectively), compared to participants in the no carotid plaque, eGFR ≥ 60 ml/min, and proteinuria <trace group. However, female participants did not show this trend.

## Discussion

In this community-based cohort study, we investigated if the combined effect increased the risk of all-cause death and CVD. Our results showed that the joint effect of carotid plaque, eGFR < 60 ml/min and proteinuria ≥ trace significantly increased the risk of all-cause death, but not CVD, compared to participants with no carotid plaque, eGFR ≥ 60 ml/min, and <trace proteinuria. However, it was found that the combined effect of a carotid plaque, eGFR < 60 ml/min, and proteinuria not only increased the risk of all-cause death but also the risk of CVD when compared to participants who did not have carotid plaque, eGFR60 < ml/min, and proteinuria.

Several studies investigate the relationship between carotid plaque, renal dysfunction, and all-cause deaths, respectively (25–27). The majority of studies found that impaired renal function is independently and significantly associated with an increased risk of mortality (28, 29). Meta-analysis showed that even a trace urine protein on a dipstick test was associated with an increased risk of mortality (8). Moreover, most research also confirms carotid atherosclerosis is an independent risk factor and further improves risk prediction for CVD when added to traditional risk factors (30–32). In our large population-based cohort from a Chinese community, we found that all-cause deaths increased 2.88-fold in participants with a carotid plaque, eGFR < 60 ml/min, and proteinuria within 6 years. Furthermore, in the population ≥ 50 years, the risk of CVD has further increased 2.98-fold in participants with a carotid plaque, eGFR < 60 ml/min, and proteinuria compared to participants with no carotid plaque, eGFR ≥ 60 ml/min, and proteinuria. As far as we know, this is the first study to assess the combined effects of impaired renal function and carotid atherosclerosis in a large sample of the general population. Our findings supported what cardiovascular prevention guidelines recommend (33), that targeted screening for atherosclerosis in those with CKD, as well as early intervention to halt the progression of atherosclerosis or renal impairment, can reduce the risk of all-cause death.

Carotid plaque, eGFR < 60 ml/min, and proteinuria ≥ trace were not associated with an increased risk of CVD in our cohort study. But in the age group of ≥ 50 years, we found that a carotid plaque, eGFR < 60 ml/min, and proteinuria ≥ trace was associated with a 1.66-fold increase in the risk of CVD. The association between lower eGFR and albuminuria with CVD was consistent with investigations from previous studies in various populations. A study in the non-Chinese population had shown that eGFR and albuminuria were predictors of CVD (34). Wang et alconfirmed these findings in a cohort of 11,940 Caucasian and 16,451 African–American diabetes patients, reporting that eGFR decline (< 60 ml/min per 1.73 m^2^) was associated with a 35 and 25% increase in the risk of stroke and coronary heart, respectively, after a 6.1–6.8 year follow up (34). Takashi Wada et al. (35) demonstrated that the presence of microalbuminuria increases the risk of cardiovascular outcomes in Japanese diabetic patients. These findings could be explained by similarities in CKD pathophysiology, age, and atherosclerosis. Risk factors for cardiovascular events such as increased levels of procoagulant biomarkers and endothelial dysfunction are associated with both reduced kidney function (36, 37) and atherosclerosis (38, 39). These factors may act synergistically to increase the risk of cardiovascular events compared with CKD or atherosclerosis alone. Furthermore, multiple cardiovascular risk factors cluster at an age ≥ 50 years. Many guidelines also suggested that patients with CKD aged ≥ 50 years be treated with a low to moderate dose of statins, regardless of their LDL cholesterol level (40, 41), and that their systolic and diastolic blood pressures be kept at an ideal level (42). Studies have found that aerobic exercise (43), limiting sodium intake (44), and a Mediterranean diet (45) are beneficial to preventing the progression of arteriosclerosis; hence people with renal impairment and carotid plaques should be encouraged to adopt a healthier lifestyle at an age ≥ 50 years.

Our analysis provides new information on the association by type of CVD, showing higher odds for MI than cerebral infarction at the age of 50 years, and shows that renal impairment and carotid plaque are associated with an increased risk of CVD. In the USA, different races, ethnicities, and subgroups experience disparities regarding MI (46). Due to the fact that the majority of our participants were Asian, we hypothesized that racial disparities might affect the results of the data. Our findings suggest that a combination of renal impairment and carotid plaque could provide useful prognostic information for identifying people who are most at risk for future MI events.

The mechanisms potentially underpinning our result that renal impairment, carotid plaques, and CVD share common cardiovascular risk factors include lipids (47) hypertension and diabetes (48) and may be amplified in patients with both factors. Other possible factors include renin-angiotensin-aldosterone system activation (49), inflammation, and oxidative stress (50). These factors may contribute to the progression of CVD.

Our research has some limitations. Firstly, the population in North China was primarily professional men. The results have limited extrapolation. Secondly, in the Kailuan Study, albuminuria was not measured at baseline, and proteinuria was evaluated only using dipsticks. As a result, we can not rule out the possibility of bias due to CKD misclassification. Finally, the study follow-up time was short (3.94 ± 2.02 years), and the final outcome may not yet have occurred.

## Conclusion

In a population-based cohort, we confirmed with our findings that the combination of carotid plaques and renal impairment may increase the risk of all-cause death and CVD to a much higher level than either factor alone in people ≥ 50 years old. We argue the case that screening individuals with CKD for carotid plaque may help to improve risk stratification and reduce the risk of cardiovascular disease and all-cause death.

## Data availability statement

The original contributions presented in this study are included in the article/Supplementary material, further inquiries can be directed to the corresponding authors.

## Ethics statement

This study was approved by the Ethics Committees of Kailuan General Hospital (Tangshan, China) and Beijing Tiantan Hospital (Beijing, China). The patients/participants provided their written informed consent to participate in this study.

## Author contributions

WL and WB carried out the studies, participated in collecting data, and drafted the manuscript. SC and CM participated in the statistical analysis. XZ, XLi, and YF drafted the figures and legends. XLu helped with revising the manuscript. SW and JH designed the outline of the topic and revised the manuscript. All authors read and approved the final manuscript.
