# Supplementary material for: Joint effects of carotid plaques and renal impairment on the risk of cardiovascular disease and all-cause death in a community-based population: The Kailuan cohort study
Source: Front Cardiovasc Med. 2022 Nov 17;9:943718. doi: 10.3389/fcvm.2022.943718 (PMC9712795; doi:10.3389/fcvm.2022.943718)
Supplement: Supplementary file 1 [file Data_Sheet_1.docx]

**Supplementary table 1. Stratified analysis of the baseline characteristics of the study population.**

|  | | No carotid plaque,  eGFR≥60ml/min, and Proteinuria<trace  (*n* = 12602) | | No carotid plaque,  eGFR<60ml/min, and Proteinuria ≥trace  (*n* = 922) | Carotid plaque,  eGFR ≥60ml/min, and Proteinuria<trace  (*n* = 6023) | Carotid plaque,  eGFR<60ml/min, and Proteinuria ≥trace  (*n* = 869) | All participants  (*n* = 20416) | *P*-value for trend |
| --- | --- | --- | --- | --- | --- | --- | --- | --- |
| Age | |  |  |  |  |  |  | |
| All, mean ± SD | | 50.46±9.15 | 54.20±11.85 | 61.89±10.50 | 66.14±10.37 | 56.18 ± 8.32 | <0.001 | |
| Male, n (%) | | 9590(76.1%) | 638 (69.2%) | 5037 (83.6%) | 712(81.9%) | 15977(78.3%) | <0.001 | |
| Smoking, n (%) | | 5091 (40.4%) | 308 (33.4%) | 2632(43.7%) | 353 (40.6%) | 8390 (41.1%) | <0.001 | |
| drinking, n (%) | | 4196 (33.3%) | 274(29.8%) | 2144 (35.6%) | 277 (31.9%) | 6900 (33.8%) | <0.001 | |
| SBP | |  |  |  |  |  |  | |
| <130 mmHg, n (%) | | 4738(37.6%) | 261 (28.4%) | 1445 (24.0%) | 148 (17.1%) | 6594 (32.3%) | <0.001 | |
| 130–139 mmHg, n (%) | | 3642 (28.9%) | 214 (23.2%) | 1258 (20.9%) | 139 (16.0%) | 5247 (25.7%) | <0.001 | |
| 140–159 mmHg, n (%) | | 3137 (24.9%) | 264 (28.7%) | 2035 (33.8%) | 295 (34.0%) | 5736 (28.1%) | <0.001 | |
| 160–179 mmHg, n (%) | | 819 (6.5%) | 120 (13.1%) | 939 (15.6%) | 186 (21.5%) | 2062 (10.1%) | <0.001 | |
| ≥180 mmHg, n (%) | | 264 (2.1%) | 61 (6.7%) | 343 (5.7%) | 99 (11.4%) | 755 (3.7%) | <0.001 | |
| <130 mmHg, mean ± SD | | 117.29±8.61 | 116.34±9.25 | 117.54±8.16 | 118.55±7.71 | 149.95±12.63 | 0.001 | |
| 130–139 mmHg, mean ± SD | | 133.87±3.07 | 133.40±3.35 | 133.31±3.21 | 133.13±3.24 | 117.33±8.52 | 0.057 | |
| 140–159 mmHg, mean ± SD | | 147.33±5.59 | 147.24±6.11 | 147.40±5.71 | 148.01±5.49 | 133.70±3.12 | ＜0.001 | |
| 160–179 mmHg, mean ± SD | | 166.54±5.68 | 166.55±5.97 | 166.47±5.89 | 167.59±6.31 | 147.38±5.65 | 0.257 | |
| ≥180 mmHg, mean ± SD | | 188.18±8.54 | 187.25±6.85 | 189.24±12.35 | 189.16±10.29 | 188.70±10.51 | 0.410 | |
| All, mean ± SD | | 134.63±18.13 | 140.00±21.87 | 143.43±20.94 | 146.89±22.32 | 142.32 ± 20.62 | <0.001 | |
| DBP | |  |  |  |  |  |  | |
| <85 mmHg, n (%) | | 7208 (57.2%) | 422 (45.8%) | 3162(52.5%) | 414 (47.7%) | 11208 (54.9%) | <0.001 | |
| 85–89 mmHg, n (%) | | 1978 (15.7%) | 109 (11.9%) | 722 (12.0%) | 78(9.0%) | 2899 (14.2%) | <0.001 | |
| 90–99 mmHg, n (%) | | 2293 (18.2%) | 226 (24.6%) | 1457 (24.2%) | 206 (23.8%) | 4185(20.5%) | <0.001 | |
| 100–109 mmHg, n (%) | | 907 (7.2%) | 121 (13.2%) | 560 (9.3%) | 128 (14.8%) | 1714 (8.4%) | <0.001 | |
| ≥110 mmHg, n (%) | | 214 (1.7%) | 41 (4.5%) | 126 (2.1%) | 41 (4.8%) | 428 (2.1%) | <0.001 | |
| <85 mmHg, mean ± SD | | 75.99±6.12 | 76.55±5.88 | 76.12±6.19 | 75.87±6.84 | 76.04±6.16 | 0.246 | |
| 85–89 mmHg, mean ± SD | | 86.92±1.26 | 87.00±1.36 | 86.95±1.28 | 87.02±1.19 | 86.93±1.27 | 0.804 | |
| 90–99 mmHg, mean ± SD | | 92.96±2.88 | 92.63±3.02 | 92.52±2.80 | 92.45±2.84 | 92.76±2.86 | ＜0.001 | |
| 100–109 mmHg, mean ± SD | | 102.12±2.67 | 101.88±2.43 | 101.98±2.69 | 102.28±3.04 | 102.07±2.69 | 0.524 | |
| ≥110 mmHg, mean ± SD | | 114.64±6.26 | 115.52±6.98 | 115.61±7.25 | 116.94±5.63 | 115.24±6.58 | 0.188 | |
| All, mean ± SD | | 83.68±10.79 | 87.11±11.27 | 85.09±11.25 | 85.14±13.35 | 83.68±10.79 | 0.004 | |
| FBG | |  |  |  |  |  |  | |
| <6.1 mmol/L, n (%) | | 9779 (77.6%) | 609 (66.1%) | 4029 (66.9%) | 499 (57.5%) | 14924 (73.1%) | <0.001 | |
| 6.1–6.99 mmol/L, n (%) | | 1625 (12.9%) | 138 (15.0%) | 975(16.2%) | 127 (14.7%) | 2878(14.1%) | <0.001 | |
| ≥7.0 mmol/L, n (%) | | 1197 (9.5%) | 174 (18.9%) | 1017 (16.9%) | 241 (27.8%) | 2633 (12.9%) | <0.001 | |
| <6.1 mmol/L, mean ± SD | | 5.23±0.46 | 5.17±0.50 | 5.23±0.47 | 5.23±0.51 | 5.23±0.47 | 0.037 | |
| 6.1–6.99 mmol/L, mean ± SD | | 6.42±0.24 | 6.47±0.26 | 6.44±0.25 | 6.47±0.25 | 6.43±0.25 | 0.021 | |
| ≥7.0 mmol/L, mean ± SD | | 9.36±3.22 | 10.01±3.12 | 9.53±3.26 | 10.13±3.13 | 9.54±3.22 | 0.001 | |
| All, mean ± SD | | 5.81±1.66 | 5.97±2.07 | 6.23±2.19 | 6.16±1.96 | 5.81±1.66 | <0.001 | |
| LDL-C | |  |  |  |  |  |  | |
| <4.1 mmol/L, n (%) | | 11669(92.6%) | 556 (92.9%) | 5402 (89.7%) | 760 (87.5%) | 18701 (91.6%) | <0.001 | |
| ≥4.1 mmol/L, n (%) | | 933 (7.4%) | 66 (7.1%) | 621(10.3%) | 109(12.5%) | 1715(8.4%) | <0.001 | |
| <4.1 mmol/L, mean ± SD | | 2.76±0.65 | 2.66±0.66 | 2.78±0.67 | 2.72±0.68 | 2.76±0.66 | <0.001 | |
| ≥4.1 mmol/L, mean ± SD | | 4.62±1.67 | 4.60±1.04 | 4.68±1.02 | 4.63±1.31 | 4.64±1.39 | 0.345 | |
| All, mean ± SD | | 2.92 ± 1.05 | 2.82 ± 0.81 | 3.00 ± 0.92 | 2.98 ± 0.94 | 2.99 ± 0.97 | <0.001 | |
| HDL-C | |  |  |  |  |  |  | |
| <1.0 mmol/L, n (%) | | 1149 (9.1%) | 107(11.6%) | 481 (8.0%) | 91 (10.5%) | 1828 (9.0%) | 0.003 | |
| ≥1.0 mmol/L, n (%) | | 11453(90.9%) | 815(88.4%) | 5542(92.0%) | 778 (89.5%) | 18588 (91.0%) | 0.247 | |
| <1.0 mmol/L, mean ± SD | | 0.89±0.10 | 0.85±0.15 | 0.88±0.09 | 0.89±0.09 | 0.88±0.10 | 0.008 | |
| ≥1.0 mmol/L, mean ± SD | | 1.49±0.45 | 1.47±0.35 | 1.55±0.89 | 1.52±0.38 | 1.51±0.61 | <0.001 | |
| All, mean ± SD | | 1.44 ± 0.48 | 1.38 ± 0.36 | 1.51 ± 0.91 | 1.45 ± 0.40 | 1.47 ± 0.69 | <0.001 | |
| TC | |  |  |  |  |  |  | |
| <6.2 mmol/L, n (%) | | 11215 (89.0%) | 802 (87%) | 5035(83.6%) | 703 (81.0%) | 17761(87.0%) | 0.456 | |
| ≥6.2 mmol/L, n (%) | | 1387 (11.0%) | 120(13.0%) | 988 (16.4%) | 166(19.0%) | 2655(13.0%) | <0.001 | |
| <6.2 mmol/L, mean ± SD | | 4.79±0.74 | 4.74±0.76 | 4.91±0.75 | 4.88±0.79 | 4.83±0.74 | <0.001 | |
| ≥6.2 mmol/L, mean ± SD | | 6.87±2.13 | 7.02±0.97 | 7.10±3.22 | 7.09±1.02 | 6.98±2.52 | <0.001 | |
| All, mean ± SD | | 5.15 ± 1.06 | 5.05 ± 1.05 | 5.28 ± 1.79 | 5.30 ± 1.21 | 5.21 ± 1.40 | <0.001 | |
| BMI | |  |  |  |  |  |  | |
| <24 kg/m2, n (%) | | 4801(38.1%) | 266(28.9%) | 2210 (36.7%) | 280 (32.2%) | 7553 (37.0%) | <0.001 | |
| 24–27.9 kg/m2, n (%) | | 5545 (44.0%) | 419 (45.4%) | 2807 (46.6%) | 401 (46.2%) | 9168 (44.9%) | <0.001 | |
| ≥28 kg/m2, n (%) | | 2256 (17.9%) | 237 (25.7%) | 1006(16.7%) | 188 (21.6%) | 3695 (18.1%) | <0.001 | |
| <24 kg/m2, mean ± SD | | 21.90±1.56 | 21.95±1.61 | 21.92±1.59 | 21.92±1.65 | 21.91±1.58 | 0.945 | |
| 24–27.9 kg/m2, mean ± SD | | 25.82±1.15 | 25.97±1.16 | 25.79±1.13 | 25.78±1.17 | 25.81±1.14 | 0.023 | |
| ≥28 kg/m2, mean ± SD | | 30.24±2.25 | 30.59±2.57 | 30.09±1.96 | 30.38±2.33 | 30.23±2.20 | 0.011 | |
| All, mean ± SD | | 25.16 ± 3.37 | 25.75 ± 3.54 | 25.14±3.23 | 25.21±3.39 | 25.12 ± 3.44 | 0.488 | |
| Taking antihypertensive drug (%) | | 1071(8.5%) | 147 (15.9%) | 1223(20.3%) | 271(31.2%) | 2712 (13.3%) | <0.001 | |
| Taking lipid-lowering drug (%) | | 264 (2.1%) | 43 (4.7%) | 361 (6.0%) | 104 (12.0%) | 775 (3.8%) | <0.001 | |

Abbreviations: BMI, body mass index; SBP, Systolic blood pressure; DBP, diastolic blood pressure; FBG, fasting blood glucose; HDL-C, high-density lipoprotein cholesterol; LDL-C, low-density lipoprotein cholesterol; TC, total cholesterol. SD: standard deviation.

**Supplementary table 2. Association of carotid plaque and renal impairment with all-cause death and cardiovascular disease events in subgroups stratified according to gender.**

|  | Males (*n* = 15977) | | Females (*n* = 4439) | |
| --- | --- | --- | --- | --- |
|  | HR (95% CI) | *P*-value | HR (95% CI) | *P*-value |
| All-cause death |  |  |  |  |
| No carotid plaque,  eGFR ≥60ml/min, and Proteinuria＜trace | 1.00 (reference) |  | 1.00 (reference) |  |
| No carotid plaque,  eGFR＜60ml/min, and Proteinuria ≥trace | 1.45(1.08–1.96) | 0.013 | 1.25 (0.85–2.13) | 0.100 |
| Carotid plaque,  eGFR ≥60ml/min, and Proteinuria＜trace | 1.16 (0.93–1.45) | 0.158 | 1.38 (0.84–2.26) | 0.197 |
| Carotid plaque,  eGFR＜60ml/min, and Proteinuria ≥trace | 1.71 (1.15–2.54) | 0.008 | 1.91 (0.96–3.77) | 0.063 |
| Cardiovascular disease events^§^ |  |  |  |  |
| No carotid plaque,  eGFR ≥60ml/min, and Proteinuria＜trace | 1.00 (reference) |  | 1.00 (reference) |  |
| No carotid plaque,  eGFR＜60ml/min, and Proteinuria ≥trace | 1.35 (0.97–1.87) | 0.069 | 2.99 (1.67–5.38) | ＜0.001 |
| Carotid plaque,  eGFR ≥60ml/min, and Proteinuria＜trace | 1.15(0.97–1.35) | 0.087 | 1.63 (1.06–2.50) | 0.024 |
| Carotid plaque,  eGFR＜60ml/min, and Proteinuria ≥trace | 1.35 (1.04–1.75) | 0.022 | 1.46 (0.69–3.08) | 0.321 |
| Cerebral ischemia |  |  |  |  |
| No carotid plaque,  eGFR ≥60ml/min, and Proteinuria＜trace | 1.00 (reference) |  | 1.00 (reference) |  |
| No carotid plaque,  eGFR＜60ml/min, and Proteinuria ≥trace | 1.52 (1.03–2.24) | 0.032 | 3.26 (1.58–6.73) | 0.001 |
| Carotid plaque,  eGFR ≥60ml/min, and Proteinuria＜trace | 1.14(0.93–1.40) | 0.186 | 1.72 (1.02–2.94) | 0.043 |
| Carotid plaque,  eGFR＜60ml/min, and Proteinuria ≥trace | 1.43 (1.04–1.95) | 0.025 | 1.44 (0.57–3.62) | 0.435 |
| Myocardial infarction |  |  |  |  |
| No carotid plaque,  eGFR ≥60ml/min, and Proteinuria＜trace | 1.00 (reference) |  | 1.00 (reference) |  |
| No carotid plaque,  eGFR＜60ml/min, and Proteinuria ≥trace | 0.87 (0.40–1.88) | 0.722 | 6.10 (1.71–21.83) | 0.005 |
| Carotid plaque,  eGFR ≥60ml/min, and Proteinuria＜trace | 1.33 (0.97–1.81) | 0.073 | 2.36 (0.80–6.94) | 0.116 |
| Carotid plaque,  eGFR＜60ml/min, and Proteinuria ≥trace | 1.37 (0.82–2.28) | 0.227 | 3.44 (0.78–15.19) | 0.102 |

^§^ Cerebral ischemia or myocardial infarction. The models were adjusted for age, sex, smoking status, alcohol consumption, body mass index, fasting blood glucose, total cholesterol, low-density lipoprotein-cholesterol, high-density lipoprotein-cholesterol, systolic blood pressure, diastolic blood pressure, antihypertensive drug use, and hypoglycemic drug use. eGFR: estimated glomerular filtration rate; HR: hazard ratio.
